# Music Attenuated a Decrease in Parasympathetic Nervous System Activity after Exercise

**DOI:** 10.1371/journal.pone.0148648

**Published:** 2016-02-03

**Authors:** Tiantian Jia, Yoshiko Ogawa, Misa Miura, Osamu Ito, Masahiro Kohzuki

**Affiliations:** 1 Department of Internal Medicine and Rehabilitation Science, Tohoku University Graduate School of Medicine, Sendai, Japan; 2 Course of Physical Therapy, Faculty of Health Science, National University Corporation Tsukuba University of Technology, Tsukuba, Japan; University Zurich, SWITZERLAND

## Abstract

Music and exercise can both affect autonomic nervous system activity. However, the effects of the combination of music and exercise on autonomic activity are poorly understood. Additionally, it remains unknown whether music affects post-exercise orthostatic tolerance. The aim of this study was to evaluate the effects of music on autonomic nervous system activity in orthostatic tolerance after exercise. Twenty-six healthy graduate students participated in four sessions in a random order on four separate days: a sedentary session, a music session, a bicycling session, and a bicycling with music session. Participants were asked to listen to their favorite music and to exercise on a cycle ergometer. We evaluated autonomic nervous system activity before and after each session using frequency analysis of heart rate variability. High frequency power, an index of parasympathetic nervous system activity, was significantly increased in the music session. Heart rate was increased, and high frequency power was decreased, in the bicycling session. There was no significant difference in high frequency power before and after the bicycling with music session, although heart rate was significantly increased. Additionally, both music and exercise did not significantly affect heart rate, systolic blood pressure or also heart rate variability indices in the orthostatic test. These data suggest that music increased parasympathetic activity and attenuated the exercise-induced decrease in parasympathetic activity without altering the orthostatic tolerance after exercise. Therefore, music may be an effective approach for improving post-exercise parasympathetic reactivation, resulting in a faster recovery and a reduction in cardiac stress after exercise.

## Introduction

Physical exercise increases sympathetic activity and decreases parasympathetic activity, resulting in an increase in heart rate (HR), and the increased HR rapidly declines after the cessation of exercise. This rapid HR recovery plays an important role in avoiding excessive cardiac work after exercise. Post-exercise decrease in HR is mediated by parasympathetic reactivation [1] and sympathetic withdrawal [2]. Previous studies have reported that trained athletes display high parasympathetic reactivation [1, 3]. However, blunted parasympathetic reactivation after exercise, resulting in reduced HR recovery, has been reported in patients with chronic heart failure [1, 4] and coronary artery disease [5]. Additionally, postponement of parasympathetic reactivation after exercise has been shown to be associated with an increased risk of sudden cardiac death [6]. Thus, post-exercise parasympathetic reactivation appears to be an important mechanism with a cardioprotective effect in both healthy participants and patients with cardiovascular disease. Therefore, an approach for improving post-exercise parasympathetic reactivation is of particular interest to reduce cardiac stress after exercise.

Music has been shown to be an efficient method of modulating emotions and autonomic nervous system activity, and is potentially a low-cost and safe adjuvant for intervention and therapy [7]. Sedative music induces both high relaxation and low tension subjectively in young adults [8]. These relaxation effects of music are supported by a shift of the autonomic balance towards parasympathetic predominance in healthy adults [9]. Additionally, listening to pleasant music provokes parasympathetic activity compared with a resting condition [10]. Thus, music may modify the autonomic nervous system activity after exercise. However, the effects of music on post-exercise autonomic nervous system activity are poorly understood.

HR variability (HRV) describes the variability of heartbeat intervals. HRV analysis, which is a reliable and non-intrusive method, is commonly used to evaluate autonomic nervous system activity [11–13]. The frequency of HRV, which is obtained from HR using methods such as fast Fourier transform, autoregressive model and maximum entropy methods (MEM), contains two major components: a low-frequency power (LF; 0.04–0.15 Hz) and a high-frequency power (HF; 0.15–0.40 Hz). LF is affected by both sympathetic and parasympathetic nervous system activities, whereas HF reflects parasympathetic nervous system activity [11]. The ratio of LF and HF (L/H) is used as an index of sympathetic nervous system activity [13].

It is also well-known that blood pressure (BP) reduces following a single bout of exercise, so-called post-exercise hypotension, in most individuals [14, 15]. Exercise induces orthostatic intolerance [16–18], which is associated with a high basal sympathetic modulation of vasomotor tone in combination with a diminished orthostatic sympathetic response to resistance vessels [19]. Music may positively or negatively alter post-exercise orthostatic hypotension through the modulation of autonomic nervous system activity. However, the effects of music on post-exercise orthostatic tolerance remain unknown.

Therefore, we evaluated the effects of music on autonomic nervous system activity after exercise in the orthostatic tolerance test using the frequency domain of HRV to test the hypothesis that music attenuates sympathetic nervous activity and/or elicits parasympathetic nervous activity after exercise, without changing the orthostatic tolerance.

## Materials and Methods

### Participants

The participants were 26 healthy volunteers (12 male, 14 female) who were recruited with a poster. Mean values for age, height, weight and body mass index (BMI) were 27.9 ± 0.7 years, 166.5 ± 1.3 cm, 59.5 ± 1.8 kg and 21.3 ± 0.4 kg/m^2^, respectively. We confirmed that participants were healthy psychologically and somatically using a questionnaire, which we designed, prior to participation in the study. No participant had an illness or was taking any medication. In addition, no participants were regular smokers or exercised regularly. Informed consent was obtained from all subjects prior to participation in this study. The ethical committee from Tohoku University School of Medicine approved this study (Approval number: 2014-1-478) and the experiment was carried out in conformity with the standards set by the Declaration of Helsinki. Participants provided their written informed consent to participate in this study.

### Study procedures

**Fig 1:**
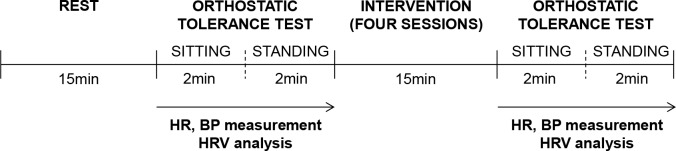
Study design. Participants rested for 15 minutes and then took the orthostatic tolerance test composed of a 2-minute sitting position and a 2-minute standing position. After the orthostatic tolerance test, participants performed 15-minute interventions: sedentary, music, bicycling and bicycling with music sessions in a random order on four separate days. After each session, participants took the orthostatic tolerance test again. HR and BP measurement and HRV analysis were performed during the orthostatic tolerance test.

The study design employed is illustrated in Fig 1. First, participants undertook an orthostatic tolerance test after a 15-minute rest. The orthostatic tolerance test was carried out as follows: participants were asked to sit quietly for 2 minutes, and then to stand up and remain standing for 2 minutes. Next, volunteers participated in 15- minute intervention. Four interventions were conducted in a random order on four separate days: 1) a sedentary session, 2) a music session, 3) a bicycling session, and 4) a bicycling with music session. After each session, participants took the orthostatic tolerance test again. In the sedentary session, subjects were asked to keep rest in the sitting position with their eyes closed. In the music session, participants were asked to remain at rest in the sitting position with their eyes closed while listening to their favorite music, which they subjectively felt to be slow, quiet and relaxing, using headphones (RP-HB400W, Panasonic Co., Osaka, Japan). The volume of the sound was up to the participants, as long as they felt comfortable. We told the participants to listen to the same music repeatedly during the intervention. In the bicycling session, participants were asked to exercise on a cycle ergometer (Monark Rehab Trainer 881E, Vansbro, Sweden) with the load of the pedals set to 60 watts for males and 40 watts for females. These values were based on a preliminary study in which participants exercised on a cycle ergometer with several different loads on the pedals, and chose the exercise intensity that they felt to be “somewhat hard”. These level of exercise intensities were equivalent to a score of approximately 13 on the rating of perceived exertion (Borg’s index[20]). In the bicycling with music session, participants were asked to exercise while listening to their favorite music, which was same as that in the music session. In the music session and the bicycling with music session, participants listened to the music during the session, not before or after the session, or during the orthostatic tolerance test.The temperature of experimental room was maintained at 23°C. All experiments were performed at the same time (between 8 a.m. and 12 a.m.).We prohibited participants from listening to music, doing intense exercise, eating food and drinking any caffeinated beverage for 3 h before the experiment. Participants were also forbidden to drink alcohol for 48 h before the experiment.

### Measurement of heart rate, blood pressure and autonomic nervous system activity

HR, systolic BP (SBP), diastolic BP (DBP) and HRV indices, indicating autonomic nervous system activity, were measured in the orthostatic tolerance test before and after each session. HR was measured by ECG, and BP was measured by upper-arm type automatic sphygmomanometer. HRV indices were measured by the software program “Kiritsu-Meijin” (Crosswell, Yokohama, Japan), which is used for the assessment of automatic reflexes in the orthostatic tolerance test. Electrocardiographic R signals were obtained at 1,000 Hz, and arrhythmias or artifacts were detected and deleted automatically by the software. We checked that all electrographic wave from were saved in the software and confirmed the accuracy of the preprocessed data. HRV indices were obtained from HR using the MEM. Since the MEM does not depend on stationary data and makes it possible to assess autonomic nervous activity based on a short-term R-R interval, it has been used to assess dynamic or phasic autonomic changes during and after exercise [21–23]. The software we used provides reliable analysis of the HRV over a minimum interval of 30 seconds. Average values for one minute just before and after standing up were used as the values in the sitting position, respectively. The coefficient of variation of the R-R interval (CVRR) was determined by dividing the standard deviation (SD) of the R-R intervals by the mean (M) R-R interval. CVRR is used as an index of overall autonomic nervous system activity [24]. For frequency analysis, HF and LF were extracted[25]. HF is an index of parasympathetic nervous system activity, and the ratio of LF to HF (L/H) is an index of sympathetic nervous system activity [26–28]. We also evaluated the change in the magnitude of autonomic nervous system activity (ΔCVRR) and sympathetic nervous system activity (ΔL/H) when the participants stood up in the orthostatic tolerance test. We calculated a component coefficient of variance HF (CCVHF; √HF/average (RR)×100), a component coefficient of variance LF (CCVLF; √LF/average(RR)×100) and the norm component coefficient of variance HF (normCCVHF; CCVHF/(CCVHF+CCVLF)×100). We also evaluated the change in the magnitude of parasympathetic nervous system activity ratio (ΔnormCCVHF) while the participants maintained the standing position in the orthostatic tolerance test.

### Statistical analysis

Data are expressed as means and standard error of means (SEM). Statistical analyses were performed using SPSS version 17.0 (SPSS Inc., Chicago, IL, USA). Data were evaluated by a two-way analysis of variance (ANOVA; session × time) with post hoc analysis employing a Students’ paired t-test between the pre- and post-intervention and between the sitting and standing position and a Bonferroni correction for multiple comparisons among the groups. Statistical significance was accepted when P-values were < 0.05.

## Results

### HR, SBP and DBP in the sitting position

Table 1 summarizes the values of HR, SBP and DBP in the sitting position before and after the interventions. HR did not change after the interventions in the sedentary session, and the post-intervention HR tended to be lower compared with the pre-intervention in the music session, but not significant (P = 0.058). On the other hand, the post-intervention HR was significantly higher compared with pre-intervention in the bicycling session and the bicycling with music session (*P* < 0.001, respectively). The effect of interventions on the post-intervention HR was significant (F (3,100) = 11.191, *P* < 0.001). The post-intervention HR in the bicycling session was significantly higher compared with the sedentary session and the music session (*P* < 0.01, respectively). Similarly, the post-intervention HR in the bicycling with music session was significantly higher compared with the sedentary session (*P* < 0.05) and the music session (*P* < 0.01). There was no significant difference in the post-intervention HR between the bicycling session and the bicycling with music session. With regard to SBP and DBP, there were no differences not only between before and after the interventions but also among the sessions.

**Table 1:** HR, SBP and DBP in the sitting position.

		Sedentary session (n = 26)	Music session (n = 26)	Bicycling session (n = 26)	Bicycling with music session (n = 26)
HR (beats/min)	Before	78.0 ± 1.7	75.8 ± 1.8	79.4 ± 1.7	77.8 ± 2.1
	After	76.7 ± 1.6	74.1 ± 2.0	88.3 ± 2.32***,††, ‡‡	85.7 ± 2.3***,†, ‡‡
SBP (mmHg)	Before	110.8 ± 2.6	109.4 ± 2.3	110.3 ± 2.3	111.0 ± 2.9
	After	109.1 ± 2.4	107.9 ± 2.1	113.3 ± 2.6	112.9 ± 3.0
DBP (mmHg)	Before	70.2 ± 1.5	72.2 ± 1.6	71.4 ± 1.4	70.8 ± 1.7
	After	69.8 ± 1.5	70.1 ± 1.4	71.8 ± 1.4	71.4 ±1.4

Data are expressed as means and SEM.

HR: heart rate; SBP: systolic blood pressure; DBP: diastolic blood pressure

*** *P <* 0.001 vs before the intervention

† *P <* 0.05

†† *P <* 0.01 vs the sedentary session

‡‡ *P <* 0.01 vs the music session.

### HRV indices in the sitting position

**Fig 2:**
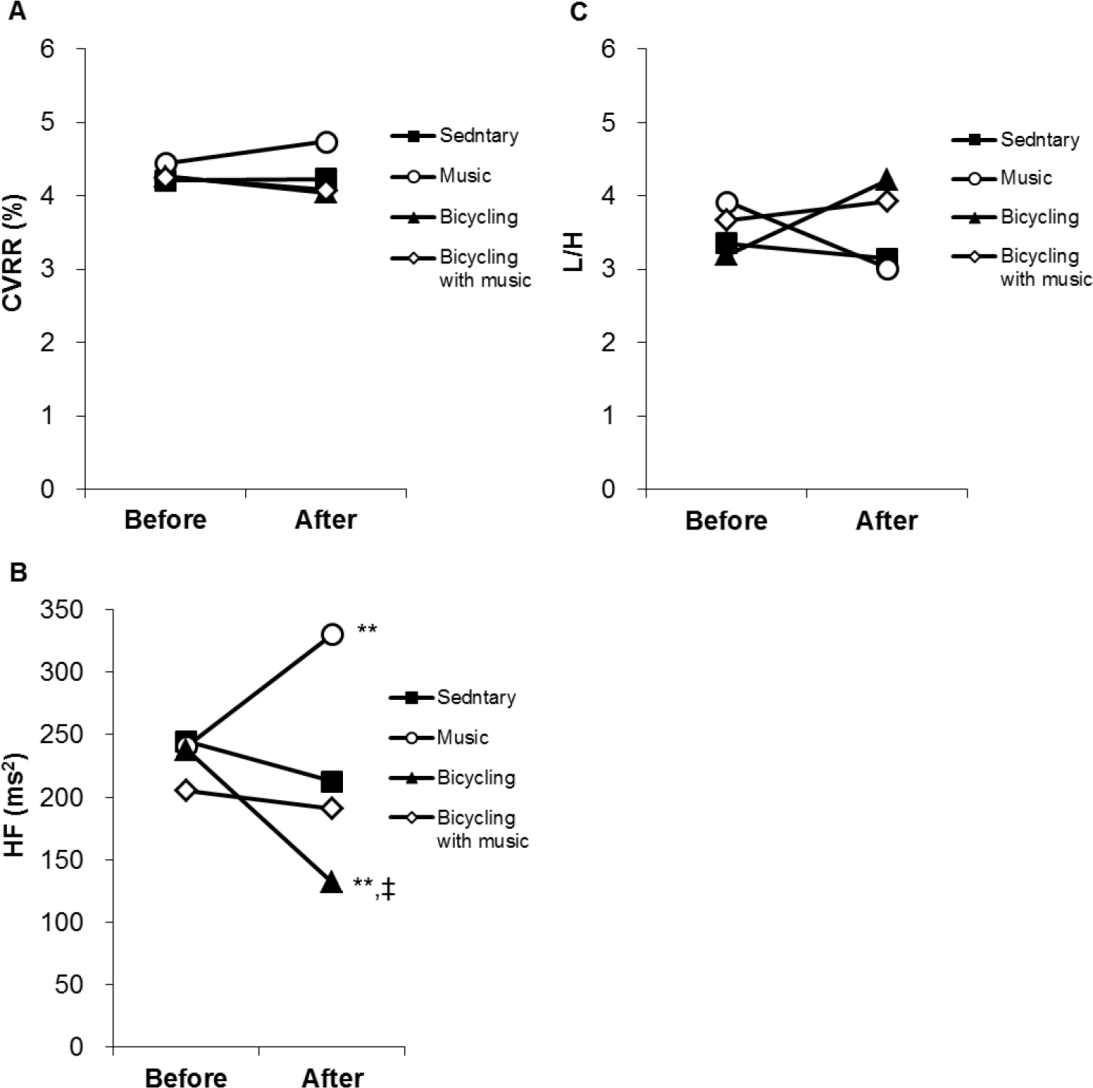
HRV indices in the sitting position. Graphs show changes in CVRR (A), HF (B) and L/H (C) in the sitting position between before and after intervention among the four sessions. CVRR: Coefficient of Variation of R-R Interval; HF: High Frequency Power; L/H: Low Frequency Power/High Frequency Power. ** *P* < 0.01 vs the sedentary session; ‡ *P <* 0.05 vs the music session. Data are expressed as means and SEM.

Fig 2 and S1 Table show the values of the HRV indices in the sitting position before and after the interventions. There were no differences in CVRR between before and after the interventions but also among the sessions (Fig 2A). The post-intervention HF was significantly higher compared with the pre-intervention in the music session (*P* < 0.01), while it was significantly lower compared with the pre-intervention in the bicycling session (*P* < 0.01) (Fig 2B). The effect of interventions on the post-intervention HF was significant (F (3,100) = 5.108, *P* < 0.01). The post-intervention HF in the bicycling session was also significantly lower compared with that in the music session (*P* < 0.05) (Fig 2B). In the bicycling with music session, there was no difference in HF between before and after the intervention (Fig 2B). With regard to L/H, there were no significant differences not only between before and after the intervention but also among the sessions (Fig 2C).

### Changes in HR and SBP when standing up

**Fig 3:**
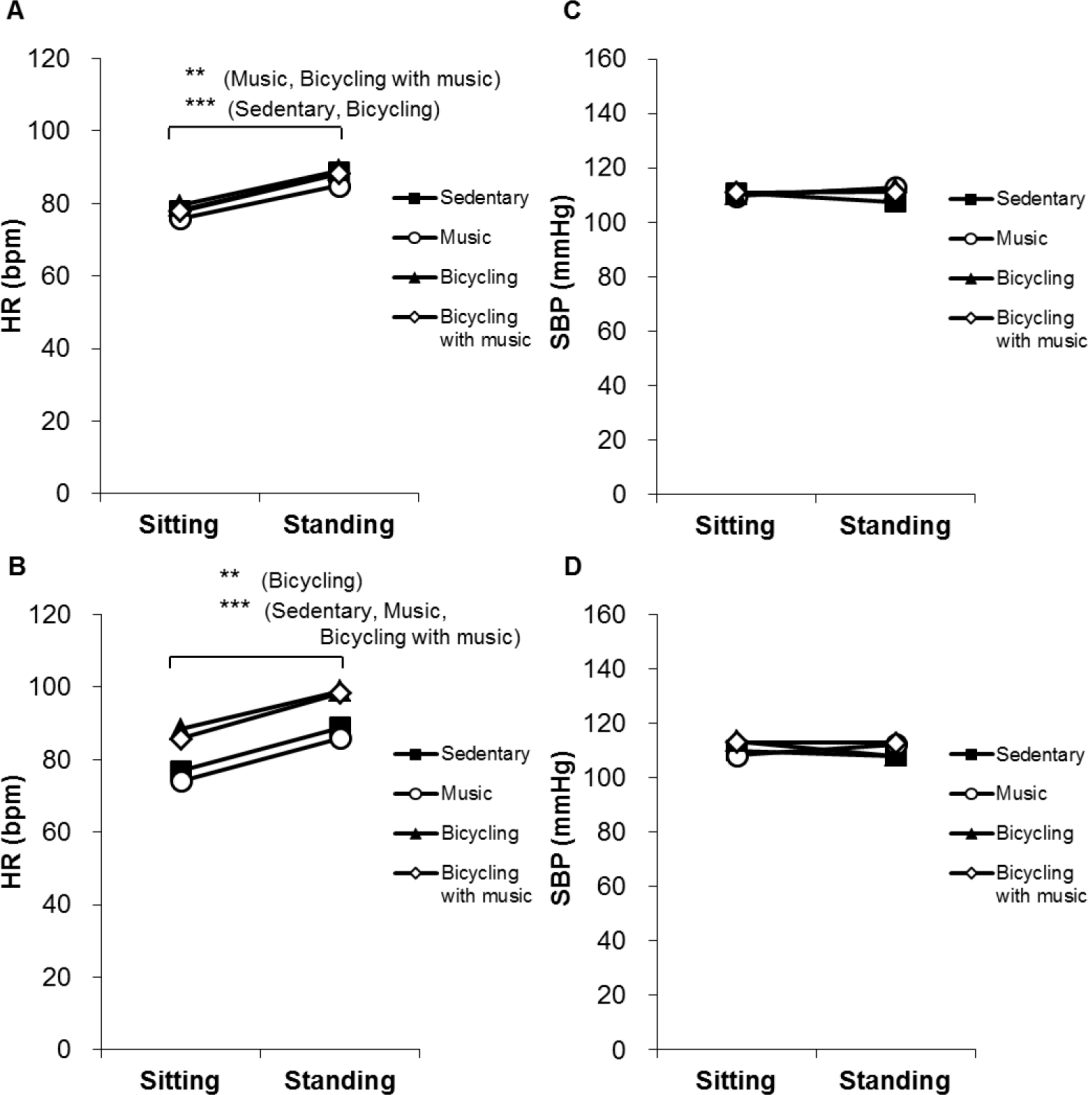
Changes in HR and SBP when standing up. Graphs show changes in HR and SBP when standing up from the sitting position in the pre-intervention (A, C) and post-intervention (B, D). HR: Heart Rate; SBP: Systolic Blood Pressure. ** *P* < 0.01, *** *P* < 0.01 vs the sitting position. Data are expressed as means and SEM.

Fig 3 shows changes in HR and SBP when standing up from the sitting position. HR was significantly increased when standing up both before and after the interventions in all sessions (Fig 3A and 3B). SBP did not change when standing up both before and after the interventions in all sessions (Fig 3C and 3D).

**Fig 4:**
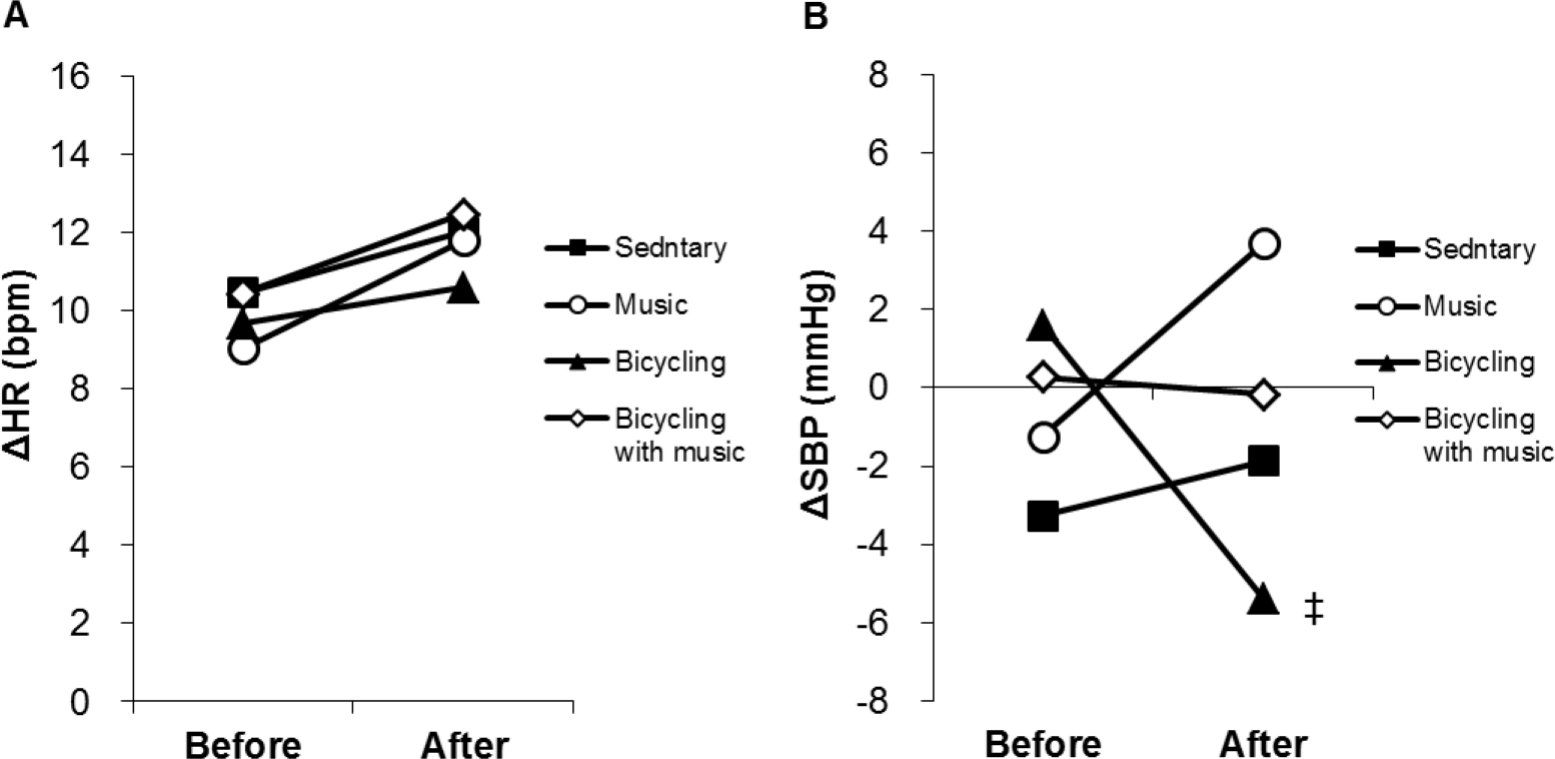
Changes in the difference of HR (ΔHR) and SBP (ΔSBP) between the sitting position and the standing position. Graphs show changes in **Δ**HR (A) and **Δ**SBP (B) between the sitting position and the standing position before and after the interventions. HR: Heart Rate; SBP: Systolic Blood Pressure; **Δ**HR: the difference of HR between in the sitting position and the standing position; **Δ**SBP: the difference of SBP between in the sitting position and the standing position. ‡ *P <* 0.05 vs the music session. Data are expressed as means and SEM.

Fig 4 shows the difference (Δ) between the sitting position and the standing position with regard to HR (ΔHR) and SBP (ΔSBP). There were no significant differences in ΔHR not only between before and after the intervention but also among the sessions (Fig 4A). There were no significant differences in ΔSBP between before and after the intervention in all sessions. The post-intervention ΔSBP in the bicycling session was significantly lower compared with the music session (Fig 4B).

### Changes in HRV indices when standing up

**Fig 5:**
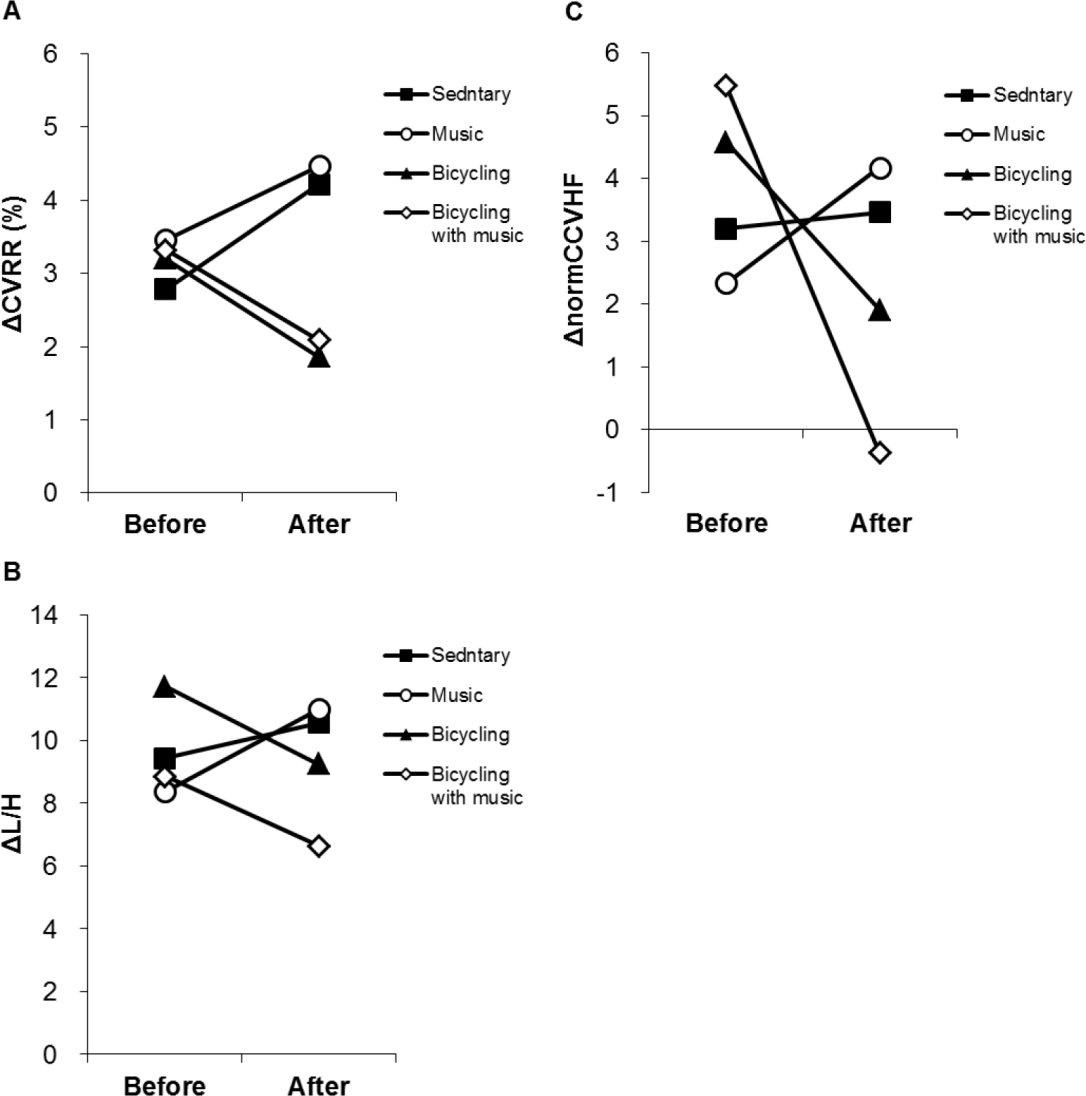
Changes in HRV indices when standing up. Graphs show changes in **Δ**CVRR (A), **Δ**L/H (B) and **Δ**normCCVHF (C) between before and after the interventions. **Δ**CVRR: change in magnitudes of autonomic nervous activity; **Δ**L/H: change in sympathetic nervous activity; **Δ**normCCVHF: change in magnitudes of a parasympathetic nervous activity ratio; CCVHF: component coefficient of variance HF. Data are expressed as means and SEM.

Fig 5 shows the values ΔCVRR, ΔL/H and ΔnormCCVHF between before and after the interventions. There were no differences in ΔCVRR between before and after the intervention in all sessions (Fig 5A). The effect of interventions on ΔCVRR was significant (F (3,100) = 11.107, *P* < 0.001). The post-intervention ΔCVRR in the bicycling session was significantly lower compared with the sedentary session and the music session (*P* < 0.001, respectively). Similarly, the post-intervention ΔCVRR in the bicycling with music session was significantly lower compared with the sedentary session (*P* < 0.01) and the music session (*P* < 0.01) (Fig 5A). With regard to ΔL/H and ΔnormCCVHF, there were no differences not only between before and after the interventions but also among the sessions (Fig 5B and 5C, respectively).

## Discussion

The present study is the first to investigate the effect of music on the autonomic nervous system activity as inferred from HRV indices in the orthostatic tolerance test after exercise. Our major findings were that the HF value was not significantly different between before and after exercise with music during exercise, while it decreased after exercise without music. Additionally, we demonstrated that changes in HR, SBP and HRV indices when standing up in the orthostatic tolerance test were not altered by either music or exercise. These results support our hypothesis that music elicits parasympathetic nervous activity after exercise, without changing the orthostatic tolerance.

The post-intervention HF was significantly increased compared with pre-intervention in the music session, although it was not changed in the sedentary session. According to the literature, HF is reported to be lower or the same during music listening compared with silence [8, 29–31], not consistent with our result. These studies, however, evaluated HF during music listening, not after listening. In contrast to these studies, the soft music increased HF after music listening in young, healthy participants [9], consistent with our result. Thus, our results suggest that music increased parasympathetic nervous system activity, at least after listening. Additionally, previous studies have shown that quiet music increases parasympathetic activity [8], particularly when listening to the favorite music of participants’ own choice [32] or when listening to the same music repeatedly [8, 33]. These factors may have induced a marked increase in parasympathetic activity after listening to music in the present study.

Post-exercise HF was significantly lower than pre-exercise in the study, consistent with a previous study that demonstrated HRV indices using the frequency analysis of HRV following exercise and recovery [34]. By contrast, HF did not change after exercise while listening to music. These results suggest that music attenuated the exercise-induced decrease in parasympathetic activity after exercise. There are few reports examining the effects of the combination of music and exercise on autonomic nervous system activity. However, in contrast to our findings, music was shown to have no influence on the exercise-induced decrease in parasympathetic activity [35, 36].It is possible that differences in the type of music listened to may contribute to these discrepancies. For example, previous studies asked participants to listen music of their choice, but the genre and tempo of the music did not matter [35, 36], while we asked participants to listen to their favorite slow and quiet music. Music consists of various components, including melody, rhythm and harmony, and this complexity may cause differences in parasympathetic nervous system activity in response to it.

L/H did not change with or without both music and exercise in the present study. There are numerous inconsistent results have been reported regarding L/H [9, 29, 31, 32, 35, 37]. Furthermore, there was no enhancement of post-exercise L/H in our study. It has previously been shown that exercise increased sympathetic nervous system activity, which remained elevated after 15 minutes of recovery [38]. However, aerobic exercise, which has an intensity lower than the anaerobic threshold, does not increase sympathetic activity, but markedly decreases parasympathetic activity [39]. As the level of exercise intensity in this study was equivalent to an approximate score of 13 on Borg’s index (i.e., moderate), sympathetic nervous system activity was not increased. Additionally, previous studies have shown that sympathetic activity is increased after exercise when listening to music before and after exercise [36], rather than during exercise. Thus, the effects of music on sympathetic nervous activity after exercise may be because of changes that occur on the initial listen. The results that music increased HF and did not change L/H after exercise suggest that music may accelerate to shift the autonomic nervous system activity towards parasympathetic dominance after exercise.

The present study examined for the first time the effect of music on orthostatic tolerance with or without exercise. Orthostasis, a disturbance of the hemodynamic conditions, evokes transient central hypovolemia, which results in a sudden drop of arterial BP. Normally, BP is restored immediately by increasing HR and peripheral vascular resistance, which is mediated neuronally and hormonally [40–42]. HR increased and SBP did not change before and after the intervention in all sessions in the present study. Both music and exercise did not significantly affect not only HR and SBP but also HRV indices in the orthostatic test. In this study, music significantly increased HF after intervention, and significantly attenuated the decrease in HF after exercise. There was the concern that a music-induced increase in HF deteriorates the orthostatic intolerance related to exercise, resulting in frequent orthostatic hypotension. The results, however, suggest that the music does not significantly alter the post-exercise orthostatic tolerance.

This study has several limitations. The participants in this study were young and healthy, and it remains unclear whether the same responses exist in older people and patients. Furthermore, we did not measure autonomic nervous system activity during exercise, and the effects of long-term combination therapy with music and exercise on autonomic nervous activity remain unknown. If the results of our study in healthy adults are true of older people and patients, we will be able to establish combination therapy with music and exercise as a new component of rehabilitation programs for them. Additional studies are required to further understand the effects of a combination of music with exercise on autonomic nervous system activity.

In conclusion, we have demonstrated that music attenuates the exercise-induced decrease in HRV indices related to parasympathetic nervous system activity, without altering the orthostatic tolerance after exercise. Music may be an effective approach for improving post-exercise parasympathetic reactivation, resulting in a faster recovery and a reduction in cardiac stress after exercise.

## Supporting Information

S1 Table Absolute and normalized values of HRV indices in the sitting position. (DOCX)
