# Supplementary material for: Music Attenuated a Decrease in Parasympathetic Nervous System Activity after Exercise
Source: PLoS One. 2016 Feb 3;11(2):e0148648. doi: 10.1371/journal.pone.0148648 (PMC4739605; doi:10.1371/journal.pone.0148648)
Supplement: S1 Table — (DOCX) [file pone.0148648.s001.docx]

**Supporting Table**

**S1 Table Absolute and normalized values of HRV indices in the sitting position**

|  | | Sedentary session  (n=26) | Music session  (n=26) | Bicycling session  (n=26) | Bicycling with music session  (n=26) |
| --- | --- | --- | --- | --- | --- |
| CVRR (%) | Before | 4.21 ± 0.27 | 4.45 ± 0.30 | 4.27 ± 0.22 | 4.25 ± 0.28 |
|  | After | 4.23 ± 0.23 | 4.74 ± 0.30 | 4.04 ± 0.29 | 4.08 ± 0.31 |
| HF (ms^2^) | Before | 244.64 ± 35.27 | 240.68 ± 36.24 | 237.76 ± 40.29 | 205.10 ± 29.54 |
|  | After | 212.52 ± 22.73 | 330.48 ± 50.57** | 132.01 ± 20.49**,‡ | 190.84 ± 44.00 |
| HF (log ms^2^) | Before | 2.26 ± 0.07 | 2.23 ± 0.09 | 2.23 ± 0.07 | 2.19 ± 0.07 |
|  | After | 2.23 ± 0.07 | 2.28 ± 0.08 | 1.98 ± 0.08‡‡ | 2.07 ± 0.09‡ |
| L/H | Before | 3.35 ± 0.96 | 3.92 ± 0.69 | 3.20 ± 0.54 | 3.67 ± 0.69 |
|  | After | 3.14 ± 0.79 | 3.01 ± 0.62 | 4.21 ± 0.75 | 3.93 ± 0.83 |

Data are expressed as means and SEM.

CVRR: Coefficient of Variation of R-R Interval; HF: High Frequency Power; L/H: Low Frequency Power/High Frequency Power

** P < 0.01 vs the sedentary session; ‡ P < 0.05, ‡‡ P < 0.01 vs the music session
